# Evidences showing wide presence of small genomic aberrations in chronic lymphocytic leukemia

**DOI:** 10.1186/1756-0500-3-341

**Published:** 2010-12-20

**Authors:** Yeong C Kim, Yong-Chul Jung, Jun Chen, Ali H Alhasan, Parawee Kaewsaard, Yanming Zhang, Shuo Ma, Steve Rosen, San Ming Wang

**Affiliations:** 1 Department of Genetics, Cell Biology & Anatomy, College of Medicine, University of Nebraska Medical Center, Nebraska, USA; 2 Northshore University Healthsystem Research Institute, Evanston, IL 60201, USA; 3 McCormick School of Engineering and Applied Science, Northwestern University, Illinois, USA; 4 Department of Medicine, University of Chicago Pritzker School of Medicine, Illinois, USA; 5 Robert H. Lurie Comprehensive Cancer Center, Northwestern University, Illinois, USA; 6 ACGT Inc., Illinois, USA; 7 School of Oceanography & Environment, Xiamen University, Xiamen, PR China; 8 Interdepartmental Biological Science Graduate Program, Northwestern University, Illinois, USA; 9 Food and Drug Administration, Bangkok, Thailand

## Abstract

**Background:**

Chronic lymphocytic leukemia (CLL) is the most common adult leukemia in the western population. Although genetic factors are considered to contribute to CLL etiology, at present genomic aberrations identified in CLL are limited compared with those identified in other types of leukemia, which raises the question of the degree of genetic influence on CLL. We performed a high-resolution genome scanning study to address this issue.

**Findings:**

Using the restriction paired-end-based Ditag Genome Scanning technique, we analyzed three primary CLL samples at a kilobase resolution, and further validated the results in eight primary CLL samples including the two used for ditag collection. From 51,632 paired-end tags commonly detected in the three CLL samples representing 5% of the *HindIII *restriction fragments in the genomes, we identified 230 paired-end tags that were present in all three CLL genomes but not in multiple normal human genome reference sequences. Mapping the full-length sequences of the fragments detected by these unmapped tags in seven additional CLL samples confirmed that these are the genomic aberrations caused by small insertions and deletions, and base changes spreading across coding and non-coding regions.

**Conclusions:**

Our study identified hundreds of loci with insertion, deletion, base change, and restriction site polymorphism present in both coding and non-coding regions in CLL genomes, indicating the wide presence of small genomic aberrations in chronic lymphocytic leukemia. Our study supports the use of a whole genome sequencing approach for comprehensively decoding the CLL genome for better understanding of the genetic defects in CLL.

## Findings

CLL (Chronic lymphocytic leukemia) is an incurable disease mainly affecting the B cell lineage in the western population, with a median age of diagnosis of 72 year old [1]. Determining the cause of CLL is crucial for understanding the acquisition and for clinical diagnosis, treatment and prognosis of CLL. Genetic factors have been linked to the etiology of CLL. Cytogenetic analyses identified chromosomal abnormalities including del11q23 affecting the *ATM *gene, tri12, del 13q14, and del17p13 affecting *TP53 *gene [2]. In addition, CGH studies found gains and losses in Xp11.2-p21 and Xq21-qter [3]. Molecular studies identified three genes: *IgVH*, *CD38 *and *ZAP-70 *that correlate with CLL prognosis [4-6]. A CLL-specific microRNA signature was also identified, suggesting that microRNA deletion could be involved in CLL [7]. SNP array studies identified 2q21.2, 6p22.1 and 18q21.1 abnormalities that follow a Mendelian inheritance pattern [8]. Whole genome association studies also identified multiple loci at 2q37.3, 8q24.21, 15q21.3 and 16q24.1 that appear to be associated with genetic susceptibility to CLL [9].

Although evidence supports the involvement of genetic factors in CLL, the frequency of genomic aberrations identified in CLL is relatively lower than those observed in the leukemias affecting other types of hematopoietic lineages [10]. This information suggests that the CLL genome is relatively intact with fewer aberrations than other types of leukemia. Alternatively, more genomic aberrations may exist in CLL but these could mainly be small lesions in the CLL genome that are difficult to detect using conventional technologies due to their limited resolution. With the rapid progress of genome sequencing technologies, enthusiasm is increasing for pursuing comprehensive detection of genomic aberrations in cancer by sequencing cancer genomes. In the case of CLL, a critical issue is to know the degree of genomic aberrations in order to justify the use of whole genome sequencing approach to analyze CLL genome. We reasoned if we can scan certain CLL genomes at sufficient high resolution and at reasonable genome coverage, we should gain first-hand information to estimate the degree of genomic aberrations in CLL.

**Figure 1:**
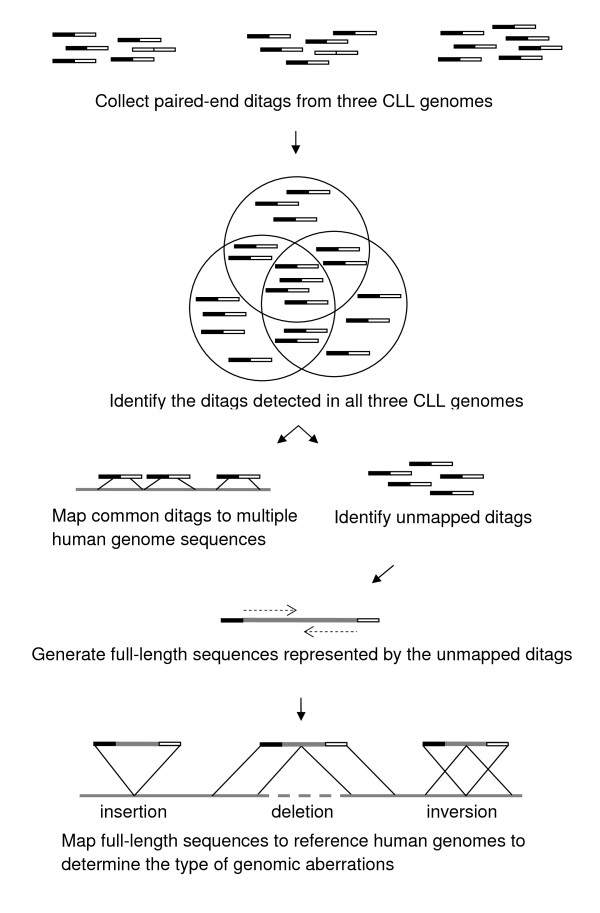
**Outline of the experimental process**. Genomic DNA samples were digested by restriction enzymes. Ditags (paired-end tags) were collected from both ends of restriction fragments and sequenced. The ditag sequences were compared to known human reference genome sequences. The unmapped ditags were used as sense and antisense PCR primers to amplify their original DNA fragments to generate full-length sequences. The sequences were mapped to reference genome sequences to determine the type of genomic aberrations.

We recently developed the DGS (Ditag Genome Scanning) technique that uses next-generation DNA sequencing technologies to collect paired-end sequences from restriction DNA fragments across a genome [11]. Using this technique, we analyzed CLL genomes. Nine samples of peripheral blood from untreated CLL patients diagnosed in Northwestern University Lurie Cancer Center and University of Chicago Medical Center were used in this study, of which three were used for paired-end tag collection, and eight including two used in paired-end tag collection were used for full-length sequencing analysis (Additional file 1: Supplemental Table S1). Informed consent was made by the patients, and the use of clinical CLL samples was approved by the institutional review board of University of Chicago and Northwestern University following institutional guidelines. The detailed experimental process followed the published protocol [11] and outlined in Figure 1. Briefly, mononuclear cells were isolated from each CLL peripheral blood or bone marrow sample by using NycoPrep™ A solution (Axis-Shield). Human genomic DNA was extracted from mononuclear cells by using QIAamp DNA Blood Kit (QIAGEN) following the manufacturer's protocol. To generate the DGS library, genomic DNA was fractionated by *HindIII *restriction digestion. The restriction fragments were dephosphorylated by CIP and cloned into pDGS-*HindIII *vector that contains two *MmeI *sites next to the *HindIII *cloning site. The genomic library was digested by *MmeI *to release two tags from the cloned DNA fragments. The tag-vector-tag fragments were then gel-purified, and re-ligated to form a ditag library. Ditags were released from the vectors by *HindIII *digestion, gel-purified, and concatemerized by using T4 DNA ligase (Promega). The concatemers at 200 to 500 bps were agarose-gel-purified and used for ditag sequencing by using a 454 GS20 sequencer (454 Life Sciences). Ditags were extracted from the resulting sequences based on the *HindIII *sites. Same ditags were combined to generate a unique ditag with the corresponding copy numbers.

To generate the reference ditag database, virtual *HindIII *restriction fragments were generated from known human genomic sequences. Two 16-bp virtual tags were extracted from the 5' and the 3' ends of each virtual fragment, and connected to form a reference ditag representing the virtual DNA fragment. The following sequences were used to extract the reference ditags:

1. Human genome reference sequences (hg18): http://hgdownload.cse.ucsc.edu/goldenPath/hg18/bigZips/

2. Human dbSNP 126: ftp://ftp.ncbi.nih.gov/snp/organisms/human_9606

3. Chimpanzee genome reference sequences (PanTro2): http://hgdownload.cse.ucsc.edu/goldenPath/panTro2/bigZips/

4. Human GM15510 fosmid paired-end sequences:

http://www.ncbi.nlm.nih.gov/Traces/trace.cgi?&cmd=retrieve&val=CENTER_PROJECT%20%3D%20%22G248%22&size=0&retrieve=Submit

5. Celera human genome sequences: http://www.ncbi.nlm.nih.gov/genomeprj/1431

6. Venter genome sequences: ftp://ftp.ncbi.nih.gov/pub/TraceDB/Personal_Genomics/Venter/

7. Watson genome sequences: ftp://ftp.ncbi.nih.gov/pub/TraceDB/Personal_Genomics/Watson/

8. Reference ditags were also extracted from *HindIII *fragments of E.coli K12 genome sequences to eliminate the ditags from E. coli DNA contaminated during library construction process.

Initial ditag mapping was performed with perfect match between experimental ditags and hg18 reference ditags. For the unmapped experimental ditags, a single-base mismatch in each single tag of the ditag was allowed to compensate for possible sequencing error or SNP. To identify the unmapped ditags related with homopolymer generated by 454 sequencing chemistry, the unmapped ditags with more than two homo-bases were stretched, e.g. AAA -> AAAA, or shortened, e.g. AAA -> AA, and mapped to reference ditags again. For the ditags remaining unmapped, they were mapped to the reference ditags of other sequence sources in the ditag reference database. The ditags remaining unmapped after these processes were defined as the unmapped ditags.

Unmapped ditag sequences were used to design sense primers and antisense (reverse/complementary) primers, with four extra bases CAGC added to the 5' end of sense primer and CGCC to the 5' end of antisense primer. Genomic DNA digested by *HindIII *was used as the templates for PCR amplification. PCR was performed with 35 cycles at 95°C 30 sec, 57°C 60 sec, and 72°C 3 min, followed by extension at 72°C for 10 min. The amplified products in each reaction were cloned into pGEM-T vector (Promega), transformed into E. coli TOP10 (Invitrogen), and plated in a single well of the 48-well Qtrays (Genetix). Four clones from each transformation were amplified by colony-PCR using M13F and M13R primers, and sequenced by Big-Dye Terminator v3.1 Cycle Sequencing Kit (ABI) using M13F primer. For the sequences that did not reach the full-length, second sequencing reactions were performed using M13R primer. To determine the genomic aberrations, each full-length sequence was mapped to hg18 using BLAT at a minimum of 90% identity as the cut-off.

The paired-end ditags were collected from three CLL samples. Genomic DNA from each sample was fractionated by *HindIII *digestion, which provides 3,561-bp resolution on average across the genome based on hg18 sequences [11]. Unique paired-end ditags of 272,193, 320,283, and 307,547 was collected from each CLL sample, covering 32%, 34% and 38% *HindIII *fragments in each CLL genome respectively. Comparing the three ditag sets shows that between 87,968 and 108,579 ditags are present between two CLL samples, and 51,632 ditags are commonly present in all three CLL samples (Table 1A). The ditags present only in individual CLL sample could be the ditags representing individual genomic differences, the ditags potentially originating from experimental artifacts, or ditags detected in one but not in others due to unsaturated ditag collection in each CLL under the sequencing scale. The 51,632 ditags detected in all three CLL samples cover 5% of genomic DNA fragments commonly detected in the three CLL genomes. In order to provide high confidence for further downstream studies, we focused on the 51,632 common ditags for further mapping analysis. We compared the 51,632 common ditags with multiple known human genome sequences, including the human genome reference sequence hg18, human SNP, human GM15510 genome sequences, chimpanzee genome sequences that are highly homologous to the humans, Watson genome sequences, and Venter genome sequences. Of the 51,632 ditags used for the mapping, 98.3% (50,799) map to hg18 that represent normal genomic fragments in the CLL genomes, 0.4% (230) are unmapped ditags that represent potential genomic aberrations commonly present in all three CLL genomes, and the remaining ditags map to other genomes that represent normal genome variations (Table 1B).

**Table 1:** Paired-end tags collected from three CLL samples

A. Ditag distribution in three CLL samples			
	**CLL sample (%)**		
	
	**1**	**2**	**3**

Total sequence reads	231,941	321,290	268,124
Total ditags	623,539	859,836	700,991
Unique ditag	272,188 (100)	320,278 (100)	307,542 (100)
Ditags common in two	99,815	99,815	
	87,968		87,968
		108,579	108,579
Ditags common in three	51,632 (19)	51,632 (16)	51,632 (17)

			
B. Mapping ditags to reference human genome sequences			

	Mapped (%)		Unmapped (%)

Total common ditags	51,632 (100)		
HG18	50,799 (98.3)		833 (1.7)
Homopolymer	22		816
Chimpanzee genome	195		616
Other human Genomes	386*		230
GM15510 genome	28		
Celera genome	165		
Venter genome	352		
Watson genome	61		

Total unmapped			230 (0.4)

*These mapped to multiple genomes was counted only once.

To determine the types of genomic aberrations for the unmapped ditags, we generated full-length sequence for the restriction DNA fragment detected by the unmapped ditags by using the "ditag-PCR" method, in which the ditag sequences were used as PCR sense and antisense primers to amplify the original DNA fragment that derived the unmapped ditag. We performed 192 reactions in eight CLL samples including two used in ditag collection and six additional CLL samples. Under the conditions that a full-length sequence must be longer than 50 bases and detected at least in the CLL used in ditag collection or at least in two additional CLL samples, 220 full-length sequences were generated from 100 unmapped ditags. Mapping the full-length sequences to hg18 identified different types of genomic aberrations caused by insertion, deletion and base change. Many of these aberrations created new *HindIII *restriction site that leads to the release of unmapped ditag, or the change of ditag sequence composition that prevents ditag mapping. These aberrations were observed in both coding and non-coding regions in CLL genome. For example, aberrations were detected in exons of NEK8, RUNX1 and MUC2 genes, and introns of 20 other genes (Table 2A, Additional file 2: Supplementary table S2). NEK8 encodes a member of the serine/threonine protein kinase family, which plays a role in cell cycle progression from G2 to M phase and is over-expressed in breast cancer [12]. A 353-base sequence converted from the unmapped ditag AAGCTTACCCTCTGGACGCCTGTATGAAGCTT maps to the last exon (Exon 15) coding for the 3' UTR of NEK8. Two *HindIII *restriction sites were inserted in the sequence that are not present in the wild-type NEK8 gene. RUNX1 is a gene involved in AML through its involvement in the t(8;21) [13]. A 434-base full-length sequence from a ditag AAGCTTCGGCCTATAG/ACAACCTAACAAGCTT was detected in all eight CLL samples, and maps to intron 3 and exon 4 of RUNX1. Analyzing the mapped region shows a T to C single-base change between the sequence and exon 4 of RUNX1 gene. Searching dbSNP reveals that this is a SNP (rs1235270). Due to the uncertainty of RUNX1 protein coding sequence itself, it is not certain if this germline SNP causes a coding amino acid change. Several bases are also changed in the mapped intron 3 of RUNX1 gene. These base changes raise an interesting question whether RUNX1 could be involved in CLL. MUC2 is a member of the MUCIN family, which codes for high molecular weight glycoproteins. The abnormalities of MUC2 is linked with colorectal and pancreas cancer [14]. A 410-base sequence derived from an unmapped ditag AAGCTTCCGGTCGGCTTCGCAGTAGAAAGCTT covers intron 29, exon 30 and intron 30 of MUC2 gene. This sequence also contains two *HindIII *restriction sites AAGCTT inserted at both its ends that do not exist in the wild-type MUC2 gene. Only three aberrations were detected in the exon of three known genes. This could be attributed to the limited genome coverage of the study and the low percentage of the exon-coding sequences in the genome. With increased genome coverage, it would be possible to identify the aberrations affecting more exons.

**Table 2:** Aberrations in exon and intron, and only present in CLL

Ditag	Full-length (bp)*	CLL Sample**								**Chr**.	Location	Aberration	Gene	
						
		1	2	3	4	5	6	7	8				Exon	Intron
A. Aberrations affecting exon and intron														
AAGCTTACCCTCTGGACGCCTGTATGAAGCTT	353	+	-	-	-	-	-	-	-	17q11.2	24093147-24093483	Insertion	NEK8	
	268	+	+	-	-	-	-	-	-	19p13.2	10331021-10331266	Insertion		TYK2
AAGCTTCGGCCTATAGACAACCTAACAAGCTT	434	+	+	+	+	+	+	+	-	21q22.12	35949034-35949460	Base change	RUNX1	
AAGCTTCCGGTCGGCTTCGCAGTAGAAAGCTT	410	-	+	-	+	-	-	-	-	11p15.5	1084558-1084956	Insertion	MUC2	MUC2
AAGCTTGAGGGTGGAGTTCCTTCTGTAAGCTT	181	-	+	+	-	-	-	-	-	2p11.2	87573183-87573358	Base change, insertion		BC070201
AAGCTTGGCCAGAGACAGGCATCATGAAGCTT	116	+	-	-	-	-	-	-	-	10q22.1	73213666-73213767	Base change		CDH23
AAGCTTGTGGACCACCGCTGTGAGTCAAGCTT	300	+	+	+	+	+	-	+	+	20p12.3	6033711-6034006	Base change		FERMT1
AAGCTTCATATGAGGATCAAAAACGAAAGCTT	283	+	+	-	+	+	+	-	-	3p14.2	61015245-61015527	Base change		FHIT
AAGCTTCTTTGTGATGCTCAGACATGAAGCTT	332	-	+	-	-	-	+	-	-	11q23.3	120263755-120264067	Base change		GRIK4
AAGCTTAGATCAGTGAGCCTACGGCGAAGCTT	605	+	+	+	+	+	+	-	+	16q22.2	69601759-69602345	Base change		HYDIN
AAGCTTCGCCGTGGGCTCACTGATCTAAGCTT	614	+	+	+	-	+	+	+	+	16q22.2	69601760-69602350	Base change, insertion		HYDIN
AAGCTTGCTGAACGCACCTGCGTGGAAAGCTT	448	+	-	+	-	-	-	+	+	5q22.2	112671099-112671541	Base change, insertion		MCC
AAGCTTGCTTCTTTGCTGATACTGGCAAGCTT	582	-	+	+	-	-	+	-	-	12q24.31	123563471-123564048	Base change, insertion		NCOR2
AAGCTTGGCGTCAATCCACACCAAAGAAGCTT	349	-	-	-	-	+	+	-	-	5q33.1	149870225-149870557	Insertion		NDST1
	1306	-	-	-	+	-	+	-	-	8q22.2	99587880-99589168	Base change, insertion		STK3
AAGCTTGAAATAAGTGCTGCATCCTGAAGCTT	163	-	+	+	-	+	-	-	-	2q32.1	182858618-182858771	Base change, insertion		PDE1A
AAGCTTTCCTAGGGAGCTGGGTGGTGAAGCTT	638	-	-	-	+	+	-	-	-	17q25.1	68849242-68849864	Insertion		SDK2
	211	+	-	-	-	-	-	-	-	9q34.11	129692280-129692481	Base change		ST6GALNAC6
AAGCTTGCAGAAGGGGAGCCAGGGTGAAGCTT	312	-	-	+	-	-	-	+	-	2p25.3	3382092-3382390	Insertion		TTC15
	187	+	-	-	-	-	+	+	-	13q12.13	26098258-26098437	Base change, insertion		WASF3
AAGCTTCAGGAAAGTCCACTAGCAAAAAGCTT	197	+	+	+	+	+	+	+	-	9p13.3	33972115-33972294	Insertion		UBAP2
	112	+	-	-	-	-	-	-	-	9p13.3	33972115-33972294	Insertion		UBAP2
	170	+	-	-	-	-	-	-	-	9p13.3	33972115-33972294	Insertion		UBAP2
AAGCTTTAATGACTGAGGGGTTCTCAAAGCTT	1147	+	+	+	-	+	-	+	-	6q25.3	157931989-157933131	Base change		ZDHHC14
AAGCTTTGAGAACCCCTCAGTCATTAAAGCTT	1134	+	+	-	-	-	-	+	-	6q25.3	157931989-157933131	Base change		ZDHHC14
AAGCTTGCACAAGGGGCCCCTTGTGCAAGCTT	691	+	-	-	-	-	-	-	-	4p16.3	2372796-2373473	Insertion		ZFYVE28
														
B. Sequences only present in CLL genome														
AAGCTTGATATCGTGATCACCTTAAGAAGCTT	332	-	+	+	-	-	-	-	-	-	-	-	-	-
AAGCTTAGATAGAGCGCAGTCAACTGAAGCTT	107	+	+	+	+	+	+	+	+	-	-	-	-	-
AAGCTTCCGGTCGGCTTCGCAGTAGAAAGCTT	159	+	+	+	+	-	+	-	-	-	-	-	-	-
AAGCTTCTCATCCTTCACCTTGGTCGAAGCTT	182***	+	-	-	-	-	-	-	-	-	-	-	-	-
	540	-	+	-	-	-	-	+	+	-	-	-	-	-
	362	-	-	-	-	-	-	+	+	-	-	-	-	-
AAGCTTGAAAAAGGTTCAGGCAAACTAAGCTT	84	+	-	-	-	-	-	-	-	-	-	-	-	-
AAGCTTGCTGAACGCACCTGCGTGGAAAGCTT	923	-	-	+	-	-	+	-	+	-	-	-	-	-
AAGCTTGGCGTCAATCCACACCAAAGAAGCTT	347	-	+	+	-	-	-	-	+	-	-	-	-	-
AAGCTTTCTTGATAAGGCTCCTACGCAAGCTT	250	-	+	-	-	-	-	-	+	-	-	-	-	-

*A full-length sequence must be detected in at least one of the two CLL sampes used in ditag collection, or at least in one other CLL samples.

** Sample #1 and 2 were used for ditag collection.

***tag1 part map to chr1:56672307-56672331

Aberrations also affect the introns of multiple genes. FHIT encodes diadenosine 5',5'''-P1,P3-triphosphate hydrolase involved in purine metabolism [15]. It is located in the common fragile site FRA3B on chromosome 3, where carcinogen-induced damage can lead to translocations in several cancers. A 283-base sequence maps to intron 8 of FHIT gene but its tag 1 contains GA to TG change. HYDIN encodes an axonemal protein; mutation of HYDIN is related to congenital hydrocephalus [16]. Two full-length sequences of 605-bp and 614-bp from two different unmapped ditags were obtained from seven CLL samples. Both sequences map to 21st intron of HYDIN. The 605-bp sequence contains CCTACGGCG in its tag 2 converted from wild-type gCcACaGCa (lowercase refers to the changed base), and the 614-bp sequence contains CGCC converted from wild-type tGCt in its tag 1 and an internal insertion. NCOR2 is a transcriptional regulator that recruits histone deacetylases to promoters [17]. A 582-base sequence maps to intron 1 of NCOR2, but its tag 1 contains an AAGC insertion, and tag 2 contains a C to T change, an AG deletion, and a T insertion. TYK2 is a member of the JAK family involving in IFN-g, IL-6, IL-10 and IL-12 signaling. Mutation in this gene is associated with hyperimmunoglobulin E syndrome [18]. A 268-base sequence maps to intron 14 of TYK2 but its tag 1 contains an AAGCTTA insertion and its tag 2 contains a TGAAGCTT insertion. Both insertions create *HindIII *restriction sites that lead to the generation of the unmapped ditag. A 197-base sequence was detected in seven CLL samples and two different sequences of 112-base and 170-base were generated from the CLL used in ditag collection. All three sequences map to *UBAP2 *located at 9p13.3, a gene involved in the ubiquitination pathway [19]. For the 197-base sequence, its 178 bases map to intron 6 of *UBAP2 *gene and the remaining 18 bases have no map, whereas the 112-base and 170-base sequences contain different insertions. Although the aberrations in many of these genes have been correlated with different types of cancer, most have not been linked with CLL.

Non-coding regions contribute to the majority of the genome, and contain important functional elements involving DNA replication, genome stability, regulation of gene expression, and coding for non-coding transcripts etc. Extensive characterization of non-coding region could provide rich candidate markers for clinical applications and identify the hotspots of genomic aberrations involving cancer development. A total of 37 sequences generated from 30 unmapped ditags mapped to the non-coding regions in the genome with various types of abnormalities (Table 3, Additional file 3: Supplemental Table S3). Although these loci are not directly located in the coding regions, many genes are located nearby the mapped locations. Of the 26 loci specifically mapped by the sequences, 15 have genes located either upstream, downstream or both within 100 kb distance. For example, a 614 base sequence maps to 5q35.1 between169443856-169444467, where DOCK2 is located 27,836 base upstream and FOXI1 is located 21028 downstream. A 398-base sequence maps to 15q26.1 between 88110782 and 88111168, where two homologous transcriptional factor genes, MESP1 and MESP2, are located 16,678-base upstream and 9,425-base downstream correspondingly. microRNA gene MIR663 are located 20,580 base upstream of 20p11.1 between 26157494-26158252 mapped by a 920-base sequence detected in seven CLL samples. Another microRNA gene MIR663B is located 10,964-base upstream of 2q21.2 between 132742087-132742356 mapped by a 290-base sequence, of which a non-coding RNA gene NCRNA00164 is located in between. The aberrations could affect the nearby genes through influencing the regulation of gene expression.

**Table 3:** Aberrations in the intergenic region

Ditag	FullL-length (bp)	CLL sample								**chr**.	Location	Aberration	Nearby genes			
						
		1	2	3	4	5	6	7	8				Upstream	Distance (bp)	Downstream	Distance (bp)
AAGCTTACTTTCTCGGTTCCATTACTAAGCTT	614	-	-	+	-	-	+	+	-	5q35.1	169443856-169444467	Base change	DOCK2	27,836	FOXI1	21,028
AAGCTTAGCCGGGCATCCTCTTTCCTAAGCTT	427	-	-	-	-	-	-	+	+	17q25.1	68688801-68689216	Base change	SSTR2	16,046	COG1	11,552
	398	-	+	+	-	-	-	-	-	15q26.1	88110782-88111168	Insertion, base change	MESP1	16,678	MESP2	9,425
AAGCTTAGTTTGGCTGCATGAGACTGAAGCTT	737	-	-	+	-	-	-	-	+	16q23.1	74682385-74683114	Base change				
AAGCTTATGATGATCCCCTGAGCTAAAAGCTT	358	+	-	-	-	-	-	-	-	1q23.3	162264795-162265148	Insertion, base change				
	264	+	-	-	-	-	-	-	-	5p15.33	2449552-2449774	Insertion				
AAGCTTCAACGATAGTTCATCATCATAAGCTT	265	-	+	+	-	-	-	-	-	16p13.3	808762-808956	Base change	PRR25	4,900	LMF1	34,679
AAGCTTCAATAGCCGAAGCCAAACTAAAGCTT	556	+	+	-	-	+	+	+	-	12q15	66234005-66234554	Base change			DYRK2	94,467
AAGCTTCACTCAGTCATATGGCATGGAAGCTT	130	-	-	-	-	-	+	+	-	10q26.3	133154300-133154419	Insertion				
AAGCTTCACTGCAGCTATAACACTGCAAGCTT	920	+	+	+	+	+	+	-	+	20p11.1	26157494-26158252	Insertion, base change	MIR663	20,580		
AAGCTTCCTCTGTACTCACATTAACGAAGCTT	892	-	+	+	-	-	+	+	-	9q12	67914079-67914963	Base change				
AAGCTTGAAATAAGTGCTGCATCCTGAAGCTT	606	+	-	-	-	-	-	-	-	1q41	220652161-220652760	Insertion, base change				
	252	-	-	+	-	-	-	-	+	20q13.13	46025615-46025861	Base change				
AAGCTTGACTCATTGCGTCTTATTCTAAGCTT	1060	+	-	-	-	-	-	-	-	9q22.31	94477651-94478404	Insertion	IPPK	5,283	BICD2	35,062
AAGCTTGCACAAGGGGCCCCTTGTGCAAGCTT	606	+	-	-	-	-	-	-	-	2q35	220216964-220217558	Insertion	SLC4A3	16,038		
AAGCTTGCAGAAGGGGAGCCAGGGTGAAGCTT	553	-	-	-	+	-	-	-	+	11q23.2	115071517-115072057	Insertion				
AAGCTTGCTGAACGCACCTGCGTGGAAAGCTT	758	-	-	-	+	+	-	-	-	13q12.3	30403963-30404714	Insertion, base change	C13orf33	25,635	C13orf26	120
	602	+	+	+	-	+	-	-	+	1q42.13	227318140-227318732	Insertion				
AAGCTTGGAGCCCTAGCCACAATTGTAAGCTT	1453	-	+	-	-	+	+	+	-	13q21.33	70285628-70287080	Base change				
AAGCTTGGCCAGAGACAGGCATCATGAAGCTT	900	+	-	+	+	-	+	-	+	6q23.2	135162469-135163363	Base change				
AAGCTTTCACTTCATTGGAGTCAGTGAAGCTT	322	+	+	+	-	+	+	+	-	13q14.12	44367511-44367832	Base change			NUFIP1	43,552
AAGCTTTCCTAGGGAGCTGGGTGGTGAAGCTT	290	-	-	+	-	-	-	+	-	2q21.2	132742087-132742356	Insertion	NCRNA00164MIR663B	10,075; 10,964		
	120	+	-	-	-	-	-	-	-	4p15.33	14280120-14280230	Insertion, base change				
AAGCTTTCCTTTTCCTTCTGCTCTTAAAGCTT	1071	+	-	+	-	-	+	-	-	6q27	164534304-164535365	Base change				
AAGCTTTGCATTGGCAGAAGCCACCAAAGCTT	1039	-	+	-	-	-	-	+	-	9q12	69920049-69921093	Base change				
AAGCTTTTAAGGGATCATGCCTCTCCAAGCTT	1534	+	-	+	-	+	+	+	-	1q21.2	148015439-148016961	Base change			FCGR1A	3,951
AAGCTTACCCTCTGGACGCCTGTATGAAGCTT	185	+	-	-	-	-	-	-	-	9q22.33	100893873-100893975	Insertion			TGFBR1	13,258

One hundred and forty seven full-length sequences converted from 57 unmapped tags map to the highly repetitive sequences in the non-coding regions. Of these sequences, 110 sequences map to the ALR/Alpha satellite sequences of the centromere, and chromosome 2, 10, and 17 are among the most frequent ones (Table 4, Additional file 4: Supplemental Table S4): 23 sequences converted from 13 unmapped tags map to the centromere of chromosome 2 at 2p11.1, 41 sequences converted from 16 ditags map to the centromere of chromosome 10 at 10q11.1, and 22 sequences converted from 6 unmapped ditags map to the centromere of chromosome 17 at 17p11.1. The presence of highly frequent aberrations in ALR/Alpha satellite sequences in these three chromosomes suggests that these could be the hot spot of genomic aberrations in CLL. Aberrations in repetitive sequences have been shown to contribute to cancer development [20]. However, it is difficult to analyze the aberrations in these highly repetitive regions using the hybridization-based approach due to the difficulty to designing specific probes. Our results show that restriction sequencing-based approach provides a useful tool to study the aberrations in these regions.

**Table 4:** Aberrations in the centromere region

Ditag	Full-length (bp)	CLL sample								**Chr**.	Location	Sequence type
					
		1	2	3	4	5	6	7	8			
AAGCTTTCATTGGGATAACAGTGTTGAAGCTT	562	-	+	+	-	-	+	+	+	2p11.1	132722630-132722850	ALR/Alpha
	893	-	+	-	-	-	-	+	-	2p11.1	91677156-91682632	ALR/Alpha
	217	+	+	-	+	-	-	+	-	2p11.1	91677835-91680254	ALR/Alpha
AAGCTTTCCAGTTAAGCTTTCTGGGGAAGCTT	1067	+	+	+	+	+	+	+	-	2p11.1	91257036-91258039	
	1002	+	-	-	-	-	-	+	-	2p11.1	91257036-91258039	
AAGCTTCTTTATGAGGAACAGTGTTGAAGCTT	216	+	+	+	+	-	-	+	-	2p11.1	91670531-91670746	ALR/Alpha
	896	+	+	-	+	-	-	-	-	2p11.1	91670531-91686712	ALR/Alpha
	901	+	-	-	-	-	-	+	-	2p11.1	91670531-91686712	ALR/Alpha
	560	+	-	-	-	+	-	-	-	2p11.1	91655191-91672448	ALR/Alpha
	2231	-	-	-	-	+	+	-	-	2p11.1	91670550-91684334	ALR/Alpha
AAGCTTCTGAGAATGCCATCCCAATGAAGCTT	686	+	-	-	-	-	-	+	-	2p11.1	91677155-91689898	ALR/Alpha
AAGCTTATTTGAGATGAAAGGAGTAGAAGCTT	1234	+	+	-	-	-	-	-	-	2p11.1	91664565-91688410	ALR/Alpha
	726	+	+	+	+	+	+	+	-	2p11.1	91676309-91680254	ALR/Alpha
AAGCTTCAACACTGTTGTTCCCAATGAAGCTT	612	-	+	+	-	-	+	-	-	2p11.1	91676431-91689428	ALR/Alpha
AAGCTTCAATGGGATGAAGAGTGTTGAAGCTT	556	+	+	+	-	+	+	+	-	2p11.1	91684461-91685014	ALR/Alpha
	894	-	-	+	-	-	-	+	-	2p11.1	91677156-91680254	ALR/Alpha
AAGCTTCAATTGGGATAACAGTGTTGAAGCTT	555	-	-	-	+	-	+	+	+	2p11.1	91677836-91680592	ALR/Alpha
AAGCTTCATTAGGGATAACAGTGTTGAAGCTT	555	+	+	-	-	-	+	-	-	2p11.1	91677156-91677709	ALR/Alpha
AAGCTTCATTGGGAACAACAGTGTTGAAGCTT	269	-	+	-	-	-	-	+	-	2p11.1	91677155-91677709	ALR/Alpha
AAGCTTCATTGGGATGGCATTCTCAGAAGCTT	685	+	-	-	-	-	-	-	-	2p11.1	91674610-91684466	ALR/Alpha
AAGCTTCTATTGGGATAACAGTGTTGAAGCTT	556	+	+	+	-	-	+	+	+	2p11.1	91672232-91680592	ALR/Alpha
	893	-	+	-	-	-	-	+	+	2p11.1	91653836-91682632	ALR/Alpha
AAGCTTGACTCATTGCGTCTTATTCTAAGCTT	1179	-	+	+	+	+	+	+	-	2p11.1	91031886-91033063	ERVL-B4-int
AAGCTTAAAACTCCTTTATGAAAAGAAAGCTT	637	+	-	-	-	-	-	-	-	10q11.1	41848823-41861037	ALR/Alpha
AAGCTTAAACTCCGTGCATCAAAAGAAAGCTT	689	+	+	-	-	-	-	+	-	10q11.1	41718813-41720661	ALR/Alpha
	1407	+	-	-	-	-	-	-	-	10q11.1	41718474-41727608	ALR/Alpha
	601	+	-	-	-	-	-	-	-	10q11.1	41718888-41720661	ALR/Alpha
AAGCTTAAACTTCTTGTATGAAAAGAAAGCTT	2067	-	+	+	-	-	-	+	-	10q11.1	41847790-41864775	ALR/Alpha
	1023	+	+	-	-	-	-	-	-	10q11.1	41718797-41729299	ALR/Alpha
	970	+	-	-	-	-	-	-	-	10q11.1	41850170-41864775	ALR/Alpha
	346	+	-	-	-	-	-	+	-	10q11.1	41718460-41719477	ALR/Alpha
AAGCTTCAACGCTGCGCTATTGAAGGAAGCTT	345	-	+	+	-	-	-	-	-	10q11.1	41726415-41729786	ALR/Alpha
	860	+	+	+	+	+	+	+	+	12p11.1	34724897-34729300	ALR/Alpha
AAGCTTCAACTCTGTCCGCCTAAAGGAAGCTT	175	-	-	-	-	+	-	+	-	10q11.1	41719301-41720316	ALR/Alpha
AAGCTTCAACTCTGTGCATTGGCCTCAAGCTT	279	+	+	+	+	+	+	+	+	10q11.1	41849321-41850275	ALR/Alpha
	622	+	+	+	+	-	+	+	-	10q11.1	41849321-41858079	ALR/Alpha
	619	-	+	-	-	-	+	+	-	10q11.1	41849321-41861137	ALR/Alpha
AAGCTTCAACTCTGTGCCGCTAAAGGAAGCTT	344	-	+	+	-	+	+	+	+	10q11.1	41718623-41720492	ALR/Alpha
	2280	+	-	-	-	-	+	-	-	10q11.1	41717944-41720994	ALR/Alpha
	1359	+	-	-	-	-	-	-	-	10q11.1	41717944-41729975	ALR/Alpha
	1190	+	-	-	-	-	-	-	-	10q11.1	41718623-41720492	ALR/Alpha
	681	+	-	-	-	-	-	-	-	10q11.1	41720147-41729975	ALR/Alpha
AAGCTTCCTTCAGAAACAAGGAGTTTAAGCTT	858	+	-	+	-	+	+	+	-	10q11.1	41718623-41720661	ALR/Alpha
AAGCTTCCTTTTAGGCCACAGAGTTGAAGCTT	348	-	+	+	-	-	-	-	-	10q11.1	41719301-41720492	ALR/Alpha
	1029	-	+	+	-	-	-	-	-	10q11.1	41847960-41861361	ALR/Alpha
	684	+	-	-	-	-	-	+	-	10q11.1	41718622-41721845	ALR/Alpha
AAGCTTCCTTTTCATACAAGGAGTTTAAGCTT	1498	-	+	-	-	-	+	-	-	10q11.1	41718461-41720661	ALR/Alpha
AAGCTTCTTTTTCATGCAAGGAGTTTAAGCTT	385	+	-	-	-	+	+	+	-	10q11.1	41718767-41719477	ALR/Alpha
	724	+	-	-	-	-	-	-	-	10q11.1	41718767-41720661	ALR/Alpha
	722	+	-	-	-	-	-	-	-	10q11.1	41847452-41858698	ALR/Alpha
AAGCTTTCCTTTAGGCCACAGAGTTGAAGCTT	1904	-	+	+	-	-	-	-	-	10q11.1	41847960-41863582	ALR/Alpha
	345	-	-	+	+	-	-	+	-	10q11.1	41720491-41722352	ALR/Alpha
AAGCTTTCTTTTTCATCAAGGAGTTTAAGCTT	386	+	-	-	-	-	-	-	-	10q11.1	41720293-41720661	ALR/Alpha
AAGCTTTGAAATCTCCCACCTAAAGGAAGCTT	408	+	-	+	+	+	+	+	-	10q11.1	41718623-41722413	ALR/Alpha
	750	-	-	+	+	-	-	-	-	10q11.1	41847563-41863586	ALR/Alpha
	1262	+	-	-	-	-	-	-	-	10q11.1	41718623-41720552	ALR/Alpha
AAGCTTTTCTTTTCATCAAGGAGTTTAAGCTT	1379	-	-	+	-	-	-	+	-	10q11.1	41718797-41720661	ALR/Alpha
	691	-	-	+	-	-	+	-	-	10q11.1	41718460-41720661	ALR/Alpha
	2041	+	-	-	-	-	-	-	-	10q11.1	41847790-41866145	ALR/Alpha
	832	+	-	-	-	-	-	-	-	10q11.1	41718460-41720661	ALR/Alpha
	347	-	-	-	+	-	+	+	-	10q11.1	41718460-41720661	ALR/Alpha
	1024	+	+	-	-	-	+	+	-	10q11.1	41718117-41720661	ALR/Alpha
AAGCTTTTGAGGCCAACACAGAGTTGAAGCTT	620	-	+	-	-	-	+	+	+	10q11.1	41849321-41855026	ALR/Alpha
	281	+	+	-	-	-	-	-	-	10q11.1	41849321-41850276	ALR/Alpha
AAGCTTCCTGTGATGATTCGAGAGAGAAGCTT	1419	+	+	-	-	+	+	-	-	17p11.1	22175465-22179262	ALR/Alpha
	576	-	-	-	-	-	+	+	+	17p11.1	22182601-22184019	ALR/Alpha
	1966	+	-	+	-	-	-	-	-	17p11.1	22175465-22186396	ALR/Alpha
	2314	-	-	+	-	-	+	-	-	17p11.1	22173089-22186396	ALR/Alpha
	234	+	+	+	+	-	+	-	+	17p11.1	22170709-22172128	ALR/Alpha
	578	+	+	+	-	-	+	-	+	17p11.1	22176307-22179262	ALR/Alpha
	1090	-	+	-	-	-	-	+	+	17p11.1	22170709-22179262	ALR/Alpha
AAGCTTTCTCTCTCGACATCACAGAGAAGCTT	641	+	-	-	-	-	-	-	-	17p11.1	22178721-22179262	ALR/Alpha
	1389	+	-	-	-	-	-	-	-	17p11.1	22175464-22184019	ALR/Alpha
	1824	+	-	-	-	-	-	-	-	17p11.1	22175464-22181640	ALR/Alpha
	579	-	+	-	-	+	+	+	-	17p11.1	22170709-22179262	ALR/Alpha
	1420	-	+	+	-	+	+	-	-	17p11.1	22180222-22184019	ALR/Alpha
AAGCTTCTCTCTCGAACATCGCAGAGAAGCTT	1091	+	-	-	-	-	+	-	+	17p11.1	22173083-22184019	ALR/Alpha
	749	-	-	+	-	-	-	-	+	17p11.1	22183291-22184019	ALR/Alpha
AAGCTTCTCTGAGATGTTCGAGAGAGAAGCTT	579	+	+	+	+	-	-	+	+	17p11.1	22181236-22184019	ALR/Alpha
	918	+	+	+	-	-	-	-	-	17p11.1	22173087-22184019	ALR/Alpha
	407	-	+	-	+	-	+	-	-	17p11.1	22174101-22184019	ALR/Alpha
	406	-	-	+	-	-	-	-	+	17p11.1	22175464-22176883	ALR/Alpha
	1087	+	-	-	-	-	-	-	-	17p11.1	22175459-22181640	ALR/Alpha
AAGCTTCTGAGAATGCTTTTCTGAAAAAGCTT	355	+	+	-	-	+	+	+	-	17p11.1	22184624-22184977	ALR/Alpha
	1037	+	-	-	-	-	-	-	-	17p11.1	22184624-22186848	ALR/Alpha
AAGCTTTGAGACCTGTCTCAGAGTTGAAGCTT	799	+	+	+	-	+	+	+	-	17p11.1	21687309-21687527	ALR/Alpha

Ten full-length sequences generated from eight unmapped ditags did not map to known human genome sequences (Table 2B. Additional file 5: Supplementary table S5). For example, a 107-base full-length sequence converted from an unmapped ditag AAGCTTAGATAGAGCGCAGTCAACTGAAGCTT was detected in all eight CLL samples. However, it does not map to the reference genome sequences. These sequences represent the DNA contents present in CLL genomes but not in normal genomes.

Through high-resolution scanning of three CLL genomes and verifying the results using full-length sequences and additional CLL genomes, our study provides evidence showing the wide presence of genomic aberrations in CLL, of which most are small lesions. Studies with increased number of CLL samples and at high genome coverage will be required to better understand the genetic aberrations in CLL. Although the study used multiple genomics databases to eliminate the changes from normal genomic polymorphism, further studies with normal DNA from the same patient will be required to fully distinguish somatic mutations from germline variations in CLL.

## Competing interests

The authors declare that they have no competing interests.

## Authors' contributions

YK, YCJ, JC, AHA, PK performed laboratory work. YZ, SM, SR provided clinical samples and data analysis, SR, SW designed the experiment. SW wrote the paper. All authors read and approved the final manuscript.

## Supplementary Material

Additional file 1 **Supplementary table S1**. CLL samples used for the study. Click here for file

Additional file 2 **Supplementary table S2**. Aberrations in exon and intron. Click here for file

Additional file 3 **Supplementary table S3**. Aberrations in the intergenic region. Click here for file

Additional file 4 **Supplementary table S4**. Aberrations in the repetitive region. Click here for file

Additional file 5 **Supplementary table S5**. Aberrations only present in CLL genomes. Click here for file
